# Non-hormonal strategies for managing menopausal symptoms in cancer survivors: an update

**DOI:** 10.3332/ecancer.2019.909

**Published:** 2019-03-11

**Authors:** Nicoletta Biglia, Valentina E Bounous, Francesco De Seta, Stefano Lello, Rossella E Nappi, Anna Maria Paoletti

**Affiliations:** 1 Division of Gynecology and Obstetrics, Department of Surgical Sciences, School of Medicine, University of Torino, Largo Turati 62, 10128 Torino, Italy; 2 Institute for Maternal and Child Health-IRCCS ‘Burlo Garofolo’, University of Trieste, via dell’Istria 65/1, 34137 Trieste, Italy; 3 Department of Woman and Child Health, Policlinico Gemelli Foundation, Largo Gemelli 1, 00168 Rome, Italy; 4 Research Center for Reproductive Medicine, Gynecological Endocrinology and Menopause, IRCCS S Matteo Foundation, Department of Clinical, Surgical, Diagnostic and Paediatric Sciences, University of Pavia, Viale Camillo Golgi 19, 27100 Pavia, Italy; 5 Department of Obstetrics and Gynecology, Department of Surgical Sciences, University of Cagliari, University Hospital of Cagliari, SS 554 km 4,500, 09042 Monserrato, Italy

**Keywords:** vasomotor symptoms, hot flushes, menopause, non-oestrogenic therapies, non-hormonal therapies

## Abstract

Vasomotor symptoms, particularly hot flushes (HFs), are the most frequently reported symptom by menopausal women. In particular, for young women diagnosed with breast cancer, who experience premature ovarian failure due to cancer treatments, severe HFs are an unsolved problem that strongly impacts on quality of life. The optimal management of HFs requires a personalised approach to identify the treatment with the best benefit/risk profile for each woman. Hormonal replacement therapy (HRT) is effective in managing HFs but it is contraindicated in women with previous hormone-dependent cancer. Moreover, many healthy women are reluctant to take HRT and prefer to manage symptoms with non-hormonal strategies. In this narrative review, we provide an update on the current available non-oestrogenic strategies for HFs management for women who cannot, or do not wish to, take oestrogens. Since isoflavones have oestrogenic properties and it is not known if they can be safely consumed by women with previous hormone-dependent cancer, they were excluded. Selective serotonin reuptake inhibitors/selective serotonin-norepinephrine reuptake inhibitors, as well as other neuroactive agents, some herbal remedies and behavioural strategies are considered.

## Introduction

Hot flushes (HFs) are the most bothersome menopause-related symptom, affecting up to 85% of menopausal women with various severity, frequency and duration [1, 2]. They first begin during the menopausal transition and they last around 7–10 years, although it is reported that some women can experience HFs for longer periods of time [3, 4]. Hormone replacement therapy (HRT) is considered the most effective treatment, but it is not indicated for all patients, such as for those with a personal history of hormone-dependent cancer or of venous thromboembolism [5, 6].

In particular, for young women diagnosed with breast cancer, who experience premature ovarian failure due to cancer treatments, severe HFs are an unsolved problem that strongly impacts on quality of life [7]. Nowadays, for hormone-dependent tumours in high-risk patients, extended therapy with adjuvant antihormonal treatment up to 10 years is suggested and for premenopausal women, the combination of ovarian suppression plus aromatase inhibitors should be considered [8] with consequent negative influence on climacteric symptoms [9].

Moreover, many women with no contraindication to HRT refuse hormonal treatment. In particular, after the publication of the Women’s Health Initiative (WHI) randomised trial in 2003, a progressive reduction in HRT prescription happened worldwide because HRT emerged as a potential risk factor for breast cancer [10]. Physicians are often reluctant in prescribing HRT for this reason, and nowadays, two-thirds of healthy women who seek treatment for menopausal symptoms will not be treated with HRT [5].

As a consequence, a spread of non-hormonal therapies is observed: data from different surveys show that around 30%–80% of women with HFs employ non-hormonal treatments [11–13]. The prevalence of complementary and alternative medicine (CAM) therapies is increasing as showed by the Study of Women’s Health Across the Nation, being 48.5% in 2002 and 80% in 2008 [11]. Moreover, data from surveys show that women prefer CAM to conventional therapies because they consider CAM natural and safe, having a positive effect on maintaining good general health and having no or mild side effects. However, women often do not inform physicians about their decision to start using CAM since they feel that healthcare providers (HCPs) lack knowledge about it and prefer to receive information from different sources (media, friends and relatives) [12–14].

For these reasons, it is important that HCPs are to be well informed about alternative remedies for menopausal symptoms.

Moreover, when discussing non-oestrogenic alternatives for menopausal symptoms, we have to keep in mind the placebo effect. Throughout the literature, the placebo effect is reported in up to 59% of the different studies on menopausal treatment. For this reason, a result which is similar to that achieved by placebo must be a good one, even if it is not significant. Moreover, menopause is a complex period of life where many physical and psychological changes interact, determining a higher susceptibility to the relationship between the woman and HCPs [14].

In this narrative review, we provide an update on the available non-oestrogenic alternatives for HFs treatment in patients who do not wish to or cannot employ HRT.

We searched through PubMed articles in an interval period (2000–2017) using the following keywords: menopause, hot flushes, climacteric symptoms, vasomotor symptoms, non-hormonal treatment. Only the publications written in English were included.

As in a narrative review, we summarised evidence from randomised controlled trials (RCTs) about non-oestrogenic alternatives for menopause-related HFs in order to broaden HCPs’ knowledge on the topic. Since isoflavones have oestrogenic properties and it is not known if they can be safely consumed by women with previous hormone-dependent cancer, they were excluded from this review. Thus, the present review merely reflects the expert opinion of the authors on addressing the various non-oestrogenic strategies to manage vasomotor menopausal symptoms and their appropriate application on a patient-by-patient basis.

## Management of vasomotor menopausal symptoms with non-hormonal strategies

### Selective serotonin reuptake inhibitors and selective serotonin-norepinephrine reuptake inhibitors

Selective serotonin reuptake inhibitors (SSRIs) and selective serotonin-norepinephrine reuptake inhibitors (SNRIs) are effective non-hormonal alternatives for vasomotor symptoms [15], reducing HFs intensity and frequency in percentages ranging from 20% to 65% [15–18].

Since HFs are thought to occur due to the changes in thermoregulation induced by oestrogen deprivation, with a consequent decrease in serotonin levels [19], the block of serotonin and norepinephrine receptors induced by SSRIs and SNRIs may oppose this imbalance.

Even if many studies have assessed the efficacy of SSRIs and SNRIs in reducing HFs [15], paroxetine salt 7.5 mg/day is the only officially Food and Drug Administration (FDA)-approved product for the treatment of moderate-to-severe vasomotor symptoms in menopausal women [20].

Among SSRIs, paroxetine, sertraline, fluoxetine and escitalopram have been studied for HFs in menopausal women, with most of the studies reporting positive results (Table 1). As regards SNRIs, duloxetine, venlafaxine and its active metabolite O-desmethylvenlafaxine have also shown a benefit in HF reduction (Table 2).

In breast cancer patients, SSRIs and SNRIs are proven to have a mild to moderate effect in reducing HFs, as assessed in a Cochrane Review on the efficacy of non-hormonal therapies for HFs in breast cancer survivors, including six RCTs on SSRIs/SNRIs (venlafaxine, paroxetine, fluoxetine and sertraline) conducted in 451 women [21].

In particular, as regards the use of SSRIs for treating HFs in breast cancer patients (Table 1), the double-blinded RCT by Loprinzi *et al* [22] showed that fluoxetine 20 mg/day was well tolerated and determined a significant but modest decrease in HFs’ score in 68 women, after 8 weeks of treatment.

Paroxetine, both the dose of 10 mg and 20 mg/day was evaluated in the RCT by Stearns *et al* [23] on 107 women with or without a history of breast cancer, of which more than 80% were breast cancer survivors mainly under tamoxifen. Both doses significantly decreased HFs, but the lower dose was less discontinued by women and even significantly improved sleep compared to placebo.

As regards SNRIs use for HFs in breast cancer survivors (Table 2), most studies focussed on venlafaxine [24, 25]. In the RCT by Loprinzi *et al* [24], 191 patients with or without previous breast cancer received placebo or venlafaxine at three different doses (37.5 mg, 75 mg and 150 mg/day). After week 4 of treatment, HFs severity decreased from baseline by 27%, 37%, 61% and 61%, respectively in the four groups, with significant results. Side effects (xerostomia, decreased appetite, nausea and constipation) were significantly more frequently reported in the venlafaxine 75 mg and 150 mg groups than in the placebo group. In the double-blind, placebo-controlled RCT by Boeckhout *et al* [25], venlafaxine 75 mg for 12 weeks significantly decreased by 41% HFs score in 38 breast cancer survivors (*p* < 0.001) compared to 17 patients who received placebo. The decrease in HFs score in the venlafaxine group was evident very soon, in 42% of cases as early as after 4 weeks of treatment (*p* < 0.01, compared to placebo). Interestingly, in the same study, a relevant placebo effect was seen (29% at 12 weeks, compared to baseline, *p* < 0.001). In the venlafaxine group, the more frequently reported side effects were nausea, constipation and severe appetite loss.

That being so, the American Cancer Society (ACS) and the American Society of Clinical Oncology (ASCO) recommend that primary care clinicians should offer SSRIs and SNRIs for HFs relief in breast cancer survivors [9].

SSRIs and SNRIs act rapidly, with a decrease in vasomotor symptoms as early as after 2 weeks of treatment [17]. Among SSRIs and SNRIs, paroxetine, citalopram and escitalopram carry the best safety profiles [16]. The more frequently reported side effects are nausea, asthenia, dizziness, xerostomia, constipation and sexual dysfunction [15]. SNRIs can increase blood pressure; therefore, this variable should be monitored in all patients [16].

Side effects, in combination with the fear of pharmacological interactions, may lead to early interruption of SSRIs/SNRIs treatment, with high dropout rates reported in the literature (up to 50% at 3 months) [26].

Due to the fast action in 2 weeks and to the safe tolerability profile, venlafaxine has been the more widely used antidepressant for HFs in clinical practice for many years. Nowadays, escitalopram is also prescribed as a first-line option for menopausal HFs. Indeed, due to its favourable tolerability profile, it is considered the antidepressant with the highest number of days of uninterrupted treatment, the best adherence to treatment and the lowest proportion of switching to other drugs [16, 26, 27].

For women with breast cancer, potential interference of antidepressants with tamoxifen has been reported since some SSRIs and SNRIs can inhibit CYP2D6 enzyme with a consequent decrease in the formation of the active metabolite from inactive tamoxifen. Among SSRIs, paroxetine and fluoxetine are the most potent inhibitors and they should be avoided during tamoxifen use; on the contrary, citalopram and escitalopram only have a limited inhibitory effect and can be used in tamoxifen users (Table 1). Among SNRIs, venlafaxine and desvenlafaxine are the safest choices while using tamoxifen [16] (Table 2).

Absolute contraindications to SSRIs and SNRIs use include previous neuroleptic and serotonin syndrome and the current use of monoamine oxidase inhibitors [17]. The North American Menopause Society (NAMS) suggests caution also in subjects affected by other conditions, such as bipolar disease, uncontrolled seizures, liver or kidney insufficiency, hypertension for SNRIs users or concurrent use of other SSRI or SNRI [16, 17].

All antidepressants need to be started at the lowest dose for 2 weeks and then the standard dose can be initiated. To stop the drug, in the same way, the lowest dose should be given for 2 weeks before ending the treatment. [16]

### Gabapentin and pregabalin

Gabapentin and pregabalin are anticonvulsant drugs able to decrease the frequency of HFs by binding to calcium channels located in the hypothalamus and, consequently, better modulating thermoregulatory activity [7, 28].

A beneficial effect of gabapentin on HFs was seen both in healthy menopausal women [29, 30] and in breast cancer survivors [31, 32] (Table 3).

Results from clinical studies performed in healthy menopausal women demonstrated a reduction of frequency and severity of HFs by around 50% [29, 30].

In breast cancer survivors, a 44%–57% decrease in HFs frequency and a 46%–67% decrease in HFs severity were reported with gabapentin in two studies on 420 and 115 breast cancer survivors, respectively [31, 32]. Furthermore, the quality of sleep improved with gabapentin 900 mg/day in these women [32].

For breast cancer patients, a Cochrane review includes gabapentin among the effective drugs in reducing with a mild to moderate effect HFs [20]. Furthermore, gabapentin is included among the suggested recommendations for HFs relief in breast cancer survivors by ACS and ASCO [9].

Pregabalin (150 to 300 mg/day) is effective in HF relief but it is less studied than gabapentin [7]. However, compared to SSRIs/SNRIs, gabapentin is as effective but has more side effects [6]. In a study on 115 breast cancer survivors using gabapentin 900 mg/day, 28.3% of the patients never started the treatment and a further 28% of them interrupted the treatment due to side effects [32].

The most common side effects on gabapentin are drowsiness, unsteadiness and dizziness [15, 30, 32], up to 50% in postmenopausal healthy women [29]. Consequently, withdrawal rates ranging from 12% to 17% are reported [29, 31]. NAMS alerts also for possible suicidal thoughts and behaviours with gabapentin and pregabalin [17].

### Clonidine

Clonidine is an anti-hypertensive alpha-adrenergic agonist, which may inhibit flushing by reducing peripheral vascular reactivity [33]. However, the exact mechanism of action is still unclear. In a systematic review and meta-analysis on non-hormonal therapies for menopausal HFs [18], clonidine significantly reduced the frequency of HFs in four out of ten trials included.

For breast cancer patients, two placebo-controlled RCTs included in a Cochrane systematic review [21] showed a moderate reduction in the frequency and severity of HFs.

Furthermore, in the double-blind, placebo-controlled RCT by Boeckhout *et al* [25], clonidine 0.1 mg/day for 12 weeks significantly decreased by 26% HFs score in 28 breast cancer survivors (*p* < 0.045) compared to 17 patients who received placebo. In the same study, another group of 38 breast cancer patients employed venlafaxine and the decline in HFs score was faster with venlafaxine than with clonidine.

However, significant side effects (xerostomia, dizziness, constipation, hypotension and potential hypertension, if suddenly interrupted) have been often reported with clonidine [17, 18] and, due to safety problems, its clinical use is poor.

### Purified pollen extract

Purified pollen extract (PPE) is a supplement sourced from pure pollen extract green climacteric (GC Fem). It is a combination of pollen and pistil extracts (PI 82), from plants of the Poaceae family, and vitamin E. The pollen and pistil extracts have high antioxidant enzyme superoxide dismutase activity [34]. Each tablet contains 40 mg of GC Fem, 120 mg of PI 82 and 5 mg of vitamin E.

Beneficial effects of PPE on vasomotor symptoms may derive from the inhibition of serotonin uptake at the synaptosomal junction, with an SSRI ‘like’ mode of action [34]. PPE does not contain any of the common phytoestrogens and does not show uterotropic-oestrogenic effects [34, 35].

Furthermore, the production technology which allows the elimination of potential allergens from PPE ensures patient safety, without any contraindications of its use in patients with pollen allergy [34].

PPE has proven clinical efficacy in the treatment of menopausal symptoms like HFs and insomnia in healthy women. A double-blind, placebo-controlled RCT on 64 postmenopausal symptomatic women showed that PPE is effective in HF treatment [34]. In the PPE group, a 65% reduction in HFs was observed, compared to 38% in the placebo group (*p* < 0.006). PPE also showed positive effects on other quality of life-related symptoms, such as dizziness, mood swings and tiredness, which often accompany vasomotor symptoms.

No data are available for PPE in women with breast cancer, but the lack of oestrogenic effect demonstrated in a preclinical study by Hellstrom *et al* [35] suggests that PPE can be a suitable option. In this study, PPE was found to contain low, subeffective concentrations of daidzin, daidzein and genistin at high-performance liquid chromatography. Genistein, formononetin and biochanin could not be detected. Moreover, PPE was tested in the same study for oestrogenic activity in the immature female rat uterotrophic bioassay and no uterine growth was seen with PPE in the high dose of 500 mg kg/day.

Moreover, in a recent *in vitro* study, PPE was neutral in the cell lines alone or in combination with oestradiol or growth factors in terms of cell proliferation and cell apoptosis, both in cells transfected with the progesterone receptor membrane component-1 (PGRMC1) or not. This is important safety data since recent experimental data revealed that oestrogens could trigger a further proliferative effect on breast cancer cells via PGRMC1, in addition to the proliferative effect via intracellularly located receptors [36].

In the clinical study by Winther *et al* [34], which employed PPE 2/day per 3 months, the evaluation of vaginal dryness and menstrual bleeding showed no change during PPE treatment. Moreover, serum measurements of the follicle stimulating hormone (FSH), E2, testosterone and sex hormone-binding globulin before and after the study period did not suggest any hormone effect of PPE.

Furthermore, an *in vitro* study demonstrated that PPE does not inhibit the CYP2D6 enzyme and thus does not interfere with tamoxifen metabolism [37].

Available data on the safety and efficacy of PPE are shown in Table 4.

### Black cohosh

Native American women have used the extract of black cohosh (*Actae racemosa* or *Cimicifugae racemosae*) for centuries as a phytotherapic cure for many different conditions. Nowadays, black cohosh is indicated only for the management of climacteric symptoms [38].

The mechanism underlying the bioactivity of black cohosh is still unclear. Selective modulation of oestrogen receptors (SERM), serotonin partial agonist mechanism, antioxidant and anti-inflammatory effects have been suggested [38, 39]. Initial studies using *in vitro* and *in vivo* assays suggested oestrogenic activity but these data have not been confirmed subsequently [38, 40].

Moreover, more recent different clinical trials showing no effect on the vaginal or endometrial thickness or on sexual hormones variation confirmed the lack of oestrogenic activity [38, 41, 42].

In the Herbal Alternatives for Menopause (HALT) double-blind, placebo-controlled RCT on 351 patients, black cohosh given up to 52 weeks determined no effects on vaginal epithelium, endometrium or sexual hormones [41].

In a large prospective open study on 400 postmenopausal women, the endometrial safety of black cohosh was assessed before and after 52 weeks of treatment, showing no increase in endometrial thickness on ultrasound and no case of endometrial hyperplasia or of serious adverse endometrial outcome [42].

RCTs showed HF reduction in healthy menopausal women and in breast cancer survivors [38]. However, a Cochrane systematic review of 16 RCTs, on 2,027 symptomatic menopausal women [43], did not show a significant difference between black cohosh and placebo in the frequency of HFs, concluding that there is insufficient evidence to support the use of black cohosh for controlling menopausal symptoms. The inconsistency of the evidence may be explained also by the heterogeneity of results among the studies due to the use of different parts of the plant or different kind of extract of black cohosh. In the review, at least five different oral preparations of black cohosh were included.

Another recent systematic review and meta-analysis, considering four RCTs on black cohosh, confirmed this data, showing that overall black cohosh was not associated with changes in the rate of HFs [44].

As regards side effects, suspected hepatotoxicity was previously reported, but a meta-analysis of five randomised, double-blind, controlled clinical trials on 1,020 women showed no evidence that black cohosh has any adverse effect on liver function [45].

The use of black cohosh in breast cancer patients is still controversial due to its SERM–like mechanism of action [46]. *In vitro* studies in MCF-7 cells during chronic use of black cohosh show that changes in the gene expression pattern are more similar to tamoxifen than to oestradiol [47]. In animal models, black cohosh given to rats for 40 weeks determines a dose-dependent reduction of breast cancer, suggesting a chemopreventive potential [48].

Furthermore, since breast density is a biomarker for breast cancer risk, different studies showed no significant modification in mammary breast density while using black cohosh [42, 49, 50]. In the 52-weeks study by Raus *et al* [42] on 400 postmenopausal women, increase in breast density was observed only in one patient who developed an invasive breast cancer unrelated to black cohosh use [49, 50]. In a prospective study on 74 postmenopausal patients, Hirschberg *et al* [49] observed no increase in breast density, assessed by mammography, and of breast cancer proliferation by Ki 67 determination through agobiopsy, both performed at baseline and after 24 weeks of black cohosh use. In the comparative study of Lundström *et al* [50] on 65 postmenopausal patients using black cohosh and 154 under HRT (oestradiol 2 mg/norethisterone acetate or tibolone) or placebo, breast density assessed by mammography performed at baseline and after 24 weeks was significantly increased with HRT use but not with placebo or black cohosh.

Only few RCTs have been performed in order to understand the use and safety of black cohosh in breast cancer patients. In the RCT by Jacobson *et al* [51], 85 breast cancer survivors, most of whom were on tamoxifen, were assigned to black cohosh or placebo. No significant differences were seen in HFs frequency and intensity with both treatments; however, in the black cohosh group, a significant improvement in sweating was observed. Interestingly, changes in blood levels of FSH and luteinizing hormone (LH) did not differ between the placebo and the black cohosh group. The RCT by Pockaj *et al* [52] failed to provide any evidence that black cohosh 40 mg/day reduces HFs more than the placebo (mean decrease in HFs score: 20% in the black cohosh group versus 27% in the placebo group, *p* = 0.53). Results from the only available RCTs performed in breast cancer patients show that there is currently a lack of evidence to support the use of black cohosh for HFs relief in breast cancer survivors [38, 53]. An observational, retrospective, cohort study on 18,861 breast cancer survivors, 1,102 of which receiving black cohosh, analysing diseases-free survival after breast cancer, showed no detrimental effect on recurrence rate [54].

Regarding tamoxifen users, a potential *in vitro* inhibition of CYP2D6 has been described, but clinical data suggest that the interaction of black cohosh with tamoxifen is unlikely [38].

Although black cohosh seems to have a good safety profile, more high-quality studies are needed to reach a definitive conclusion regarding its efficacy on HFs [43, 44, 46].

A summary of the evidence on the efficacy and safety of black cohosh on HFs relief is described in Table 5.

### Oxybutinin

Studies suggest that oxybutynin, an anticholinergic generally employed for urinary incontinence due to overactive bladder, is an effective treatment for HFs, both in healthy women [55] and in breast cancer survivors [56].

### Weight loss

Data from the WHI trial in healthy women show that weight loss determines a reduction in HFs [57]. Two RCTs confirm these findings, suggesting that weight loss is associated in overweight or obese healthy women with a reduction in HFs [58, 59]. In breast cancer survivors, prevention of weight gain after diagnosis can help in controlling HFs, whereas the role of intentional weight loss after diagnosis on vasomotor symptoms is still not defined [60].

### Yoga and exercise

A Cochrane review failed to demonstrate a positive effect of exercise and yoga on HFs [61]. However, a recent study reported that exercise training may decrease the severity of HFs [62] and in an RCT by Cramer *et al* [63], yoga was effective in reducing vasomotor symptoms in breast cancer survivors.

In a systematic review and meta-analysis of 13 RCTs on 1,306 women, yoga compared with no treatment reduced total menopausal symptoms, HFs, psychological and urogenital symptoms without serious adverse events [64]

### Cognitive behavioural therapy

Cognitive behavioural therapy (CBT) is, among mind-body techniques, an effective treatment for HFs both for healthy postmenopausal women and breast cancer survivors [17]. In the MENOS 2 trial, an RCT conducted on 140 healthy postmenopausal women, CBT significantly reduced vasomotor symptoms [65]. The MENOS 1 trial was an RCT specifically addressed to breast cancer patients in which CBT significantly reduced HFs after 9 weeks compared with usual care. The improvement was maintained at 26 weeks from randomisation and additional benefits to mood, sleep and quality of life were observed [66].

### Relaxation therapy

For breast cancer patients, a Cochrane Review assessed that relaxation therapy has a mild to moderate effect in reducing HFs [21]. Nevertheless, in healthy perimenopausal and postmenopausal women, a more recent Cochrane review concluded that evidence is insufficient to prove the effectiveness of relaxation techniques [67].

### Acupuncture

Acupuncture has been shown to reduce HFs in healthy women, compared with no treatment [68].

A meta-analysis including three systematic reviews and four RCTs assessed the effectiveness of acupuncture in reducing HF frequency and severity in peri or postmenopausal women, with improvement also in health-related quality of life items and without significant side effects [69].

However, in breast cancer patients, a Cochrane Review failed to confirm its effectiveness in reducing HFs [21]. Further data are needed to prove a positive effect of acupuncture and, eventually, to predict which subset of patients could benefit from it.

### Cooling strategies

Moreover, since temperature is a trigger of flushing, cooling strategies have also been proposed in order to reduce HFs (dressing in layers, with light, cotton clothing and standing away from sources of warming); however, the efficacy of such strategies is not supported by scientific evidence [17].

### Stellate ganglion block

Stellate ganglion block (SGB), consisting of a vertebral cervical block by local anaesthetic injection, has been proposed for HF treatment. It was suggested that SGB resets the temperature-regulating mechanisms by interrupting the connections between the central and sympathetic nervous system. Only one RCT on 40 postmenopausal women focussed on SGB for HFs [70], showing no significant difference in the overall HF frequency after SGB, while four open-label studies showed a 45%–90% reduction in HFs with SGB [7]. Larger RCTs are needed in order to evaluate its efficacy. Even if it is reported that SGB performed by skilled practitioners is safe [70], concerns related to the close proximity of critical structures may hinder the spread of this non-hormonal option for non-HF relief.

## Conclusion

In menopausal women, vasomotor symptoms, and in particular, HFs, frequently remain underdiagnosed and undertreated, with a negative impact on the patient’s quality of life. Paying more attention to the needs and preferences of menopausal women may reduce the number of untreated cases of HFs. Psychoactive agents are commonly used to treat HFs in some countries, such as the USA but are less well accepted by physicians and patients in others. Whatever the perception toward these drugs is, they are mostly used as off-label drugs and for short periods. On the other hand, CAM approaches have provided interesting data in recent studies. Among them, PPE has a confirmed non-oestrogenic effect and has been shown to be effective in decreasing HFs, night sweats, irritability and improving the quality of sleep in menopausal women, with consequent improvement in the quality of life. Black cohosh is a well-known traditional medicine treatment, which is widely used in spite of the inconsistency of data collected so far requiring further research. Recent evidence supports a positive role of physical activity in the management of HFs.

In order to manage HFs in menopausal women effectively, clinicians should identify the patient’s health profile and personal preferences and then select an individualised and safe therapy.

## Research agenda

Future research on the molecular mechanisms of flushing during menopause and on the effects of available and novel treatments will further improve the management of HFs in both healthy menopausal women and in women with contraindications to oestrogens.

Since limitations of the available evidence are in particular the imprecision or lack in the data and in the study method details, a great effort must be made to perform RCTs with large samples and with a strict methodology to allow comparison of the different strategies.

## Conflicts of interest

Anna Maria Paoletti, Francesco De Seta and Stefano Lello declare no conflict of interests. Nicoletta Biglia had a financial relationship (lecturer, member of advisory boards and/or consultant) with Gedeon Richter, Shionogi Limited and Italfarmaco. Rossella E Nappi has a financial relationship (lecturer, member of advisory boards and/or consultant) with Bayer HealthCare, Endoceutics, Gedeon Richter, HRA Pharma, MSD, Novo Nordisk, Pfizer, Shionogi and Teva. Valentina E Bounous had a financial relationship with Italfarmaco S.p.A. (medical writing) and has a financial relationship with Shionogi (medical writing, tables preparation).

## Funding

Financial support for medical writing (table preparation) and editorial services (English supervision) was provided by Shionogi.

## Figures and Tables

**Table 1.** SSRIs for HFs.

Type of drug and dose	Effectiveness	Side effects	Interactions with tamoxifen
**Paroxetine** 10–25 mg/day (7.5 mg salt is the only SSRIs/SNRIs approved for the treatment of menopausal moderate-to-severe HFs by FDA) *(first-line option for HFs)* [15, 16, 17, 21, 23]	Up to 64% HFs score reduction, improvement also of sleep	Nausea at the 20 mg dose The low dosage has less toxicity low withdrawal rate, in particular, with low doses	Potent inhibitors of CYP2D6 enzyme; they should be avoided during tamoxifen use
**Fluoxetine** 10–30 mg/day (s*econd-line option for HFs)* [16, 17, 21, 22]	24% HFs score and 19% HFs frequency reduction	18% withdrawal rate withdrawal due to more ineffectiveness of treatment rather than to side effects	
**Sertraline** 25–100 mg/day (s*econd-line option for HFs)* [16, 17, 21]	Modest effect on HFs	10% dropout rate nausea and decreased sexual function	Moderate effect on the CYP2D6 enzyme
**Citalopram** 10–20 mg/day *(first-line option for HFs)* [16, 17]	Up to 49%–55% HFs score reduction	20% withdrawal rate	Mild inhibitory effect on the CYP2D6 enzyme; they can be used in tamoxifen users
**Escitalopram** 10–20 mg/day *(first-line option for HFs)* [16, 17]	47% HFs frequency and 24% reduction	Best tolerability profile withdrawal rate of 4% nausea, weakness and drowsiness	

SSRIs = selective serotonin reuptake inhibitors; SNRIs = selective serotonin-norepinephrine reuptake inhibitors; FDA = Food and Drug Administration; HFs = hot flushes

**Table 2.** SNRIs for HFs.

Type of drug and dose	Effectiveness	Side effects	Interaction with tamoxifen
**Duloxetine** 30–120 mg/day *(first-line option for HFs)* [16]	56% HFs frequency and 62% HFs score reduction	Nausea, weakness, drowsiness, insomnia, mouth dryness and constipation	Moderate effect on the CYP2D6 enzyme
**Venlafaxine** 37.5–150 mg/day *(first-line option for HFs)* [16, 17, 21, 24, 25]	Immediate effect and strong HFs reduction, up to 30%–58% reduction in HFs frequency and 37%–61% in the HFs score	Nausea, constipation, dry mouth, headache, sleeplessness and decreased appetite	Low inhibitory effect on the CYP2D6 enzyme; they are the safest choices in tamoxifen users
**Desvenlafaxine** 100–150 mg/day *(first-line option for HFs)* [15, 16, 17, 21]	60%–66% HFs frequency and 24%–29% HFs severity reduction, already in the first week of treatment	Only in the first week of treatment (nausea, dizziness and headache)	

SNRIs = selective serotonin-norepinephrine reuptake inhibitors; HFs = hot flushes

**Table 3.** Main studies performed on gabapentin for HFs treatment.

Type of patients	Author, year of publication and type of study, number of patients (N)	Type of treatment	Type of measurement	Main results	Adverse events (AEs)
Healthy women	Guttuso *et al* [29] Randomised, double-blind, placebo-controlled trial *N* = 59	1) 900 mg oral gabapentin for 12 weeks versus placebo 2) Extension phase: gabapentin up to 2,700 mg/day	Diary for HFs severity and frequency, composite score including both	1) 45% HFs frequency and 54% HFs score reduction from baseline, compared with 29% (*p* < 0.02) and 31% (*p* < 0.01), respectively, for placebo 2) With the higher dose, further reduction of HFs (54% in HF frequency and 67% in the score)	- Somnolence, dizziness, rash - In 50% of gabapentin patients, at least one AE (versus 27.6% for placebo) - 13% withdrawal rate in the gabapentin group for AEs (versus 3% for placebo)
	Butt *et al* [30] Randomised, double-blind, placebo-controlled trial *N* = 200	900 mg gabapentin for 4 weeks	Diary for HFs severity and frequency, score including both	51% HFs score and 45.7% frequency reduction versus placebo (26.5% and 24.7%, respectively, *p* < 0.001)	More dizziness, unsteadiness and drowsiness in the gabapentin group versus placebo in the first treatment week, with later AEs reduction
Breast cancer survivors	Pandya *et al* [31] Randomised, double-blind, placebo-controlled, multi-institutional trial *N* = 420 BCSs	300 mg/d or 900 mg/d gabapentin versus placebo over 8 weeks	Diary for HFs severity, frequency and duration	44% HFs frequency and 46% severity reduction in the 900 mg gabapentin group versus placebo (15% for both, *p* < 0.0001) → gabapentin is effective in HFs control at a dose of 900 mg/day	- Withdrawal rate of 12% at 4 weeks and 17% at 8 weeks for AEs - Significant worsening of appetite
	Biglia *et al* [32] RCT *N* = 115 BCSs	Oral gabapentin 900 mg/day (*N* = 60) versus vitamin E 800 IU/day (*N* = 55) for 12 weeks	1) For HFs: daily HFs diary 2) For sleep quality: PSQI 3) For other menopausal Symptoms: MRS 4) For QoL: SF-36 Health Survey	1) HFs frequency and score decreased by 57% and 67%, respectively, (*p* < 0.05) in the gabapentin group 2) Improvement in quality of sleep (PSQI score reduction: 21.33%, *p* < 0.05).	The prescribed treatment with gabapentin was never started by 28.3% of BCSs and was interrupted by 28% of BCSs for AEs (dizziness and somnolence)

HFs = hot flushes; AEs: adverse events; BCSs = breast cancer survivors; PSQI = Pittsburgh Sleep Quality Index; MRS = Menopause Rating Scale; QoL = quality of life

**Table 4.** PPE for HFs treatment.

		Author, year of publication and type of study	Number of patients (N) and type of treatment	Type of measurement	Main results
Efficacy in healthy women		Winther *et al* [34] *Double-blind, placebo-controlled trial*	*N* = 64 PPE 2/day per 3 months	- MRS - 15 QoL parameters	- 65% HFs reduction in the PPE group versus 38% in the placebo group (*p* < 0.006) - Improvement in the QoL parameters (tiredness, dizziness, mood, libido, headache, irritability, mood swings and sensitiveness) in the PPE group compared to baseline (*p* < 0.031)
Safety in breast cancer survivors	No oestrogenic activity	Hellstrom *et al* [35] *In vitro study*		- High-performance liquid chromatography analyses of phytoestrogens in PPE - Oestrogenic activity evaluation in the immature female rat uterotrophic bioassay with PPE	- PPE in the high dose of 500 mg kg/day contains low, subeffective concentrations of daidzin, daidzein and genistin. Genistein, formononetin and biochanin could not be detected. - No uterine growth in female rats with PPE
		Seeger *et al* [36] *In vitro study*		- MCF-7 and T47D cells were transfected with PGRMC1 - Different concentrations of PPE alone and in combination with E2 or growth factor were tested - Proliferation was determined by the MTT test - Apoptosis was determined by CDD ELISA kit	PPE was neutral in the cell lines alone or in combination with E2 or growth factors in terms of cell proliferation and cell apoptosis, both in cells transfected with PGRMC1 or not
		Winther *et al* [34] *Double-blind, placebo-controlled trial*	*N* = 64 PPE 2/day per 3 months	- 15 QoL parameters - Diary of AUB - Blood samples for FSH, E2, TT, SHBG	- No changes in vaginal dryness parameter - No AUB - No change in blood levels of FSH, E2, TT, SHBG
	No interference with CYP2D6 enzyme	Goldstein *et al* [37] *In vitro study*		Test for potential inhibition of CYP2D6 enzyme by PPE at high concentrations in pooled human liver microsome with Quinidine as a reference.	Negligible inhibition of CYP2D6 with PPE (6.53% to 10.67%), whereas Quinidine completely inhibited the CYP2D6.

PPE = Purified pollen extract; MRS = Menopause Rating Scale; HFs = hot flushes; QoL = quality of life; PGRMC1 = progesterone receptor membrane component-1; MTT test = 3−(4,5-dimethylthiazol-2-yl) 2,5-diphenyl-tetrazolium bromide test; CDD = Cell death detection; FSH = follicle stimulating hormone; E2 = estradiol; TT = testosterone; SHBG = hormone-binding globulin; AUB = abnormal uterine bleeding; ELISA = enzyme-linked immunoassay

**Table 5.** Black cohosh for HFs treatment.

Black cohosh versus placebo	Outcome
HFs frequency and intensity	- No statistically significant difference in systematic reviews and meta-analysis [43, 44] - Same results in RCTs in BCSs [51, 52]
Night sweats frequency	- No statistically significant difference in systematic reviews and meta-analysis [43, 44] - In an RCT in BCSs significant improvement [51]
Menopausal symptom score (KI, GCS and MRS)	No statistically significant difference in systematic reviews and meta-analysis [43, 44]
Safety profile	- Good safety profile in the general population [38, 45] - No endometrial thickness increase [41, 42] - No detrimental effect on recurrence rate in BCSs [54] and unlikely interaction with tamoxifen [38]

KI = Kupperman Index; GCS = Green Climacteric Scale; MRS = menopause rating scale; RCT = randomised controlled trial; BCSs = breast cancer survivors
